# Polymeric Based Hydrogel Membranes for Biomedical Applications

**DOI:** 10.3390/membranes13060576

**Published:** 2023-06-01

**Authors:** Sonia Trombino, Roberta Sole, Federica Curcio, Roberta Cassano

**Affiliations:** Department of Pharmacy, Health and Nutritional Science, University of Calabria, Arcavacata, 87036 Rende, Italyroberta.sole@unical.it (R.S.)

**Keywords:** hydrogel membrane, tissue engineering, drug delivery, wound healing, biocompatibility

## Abstract

The development of biomedical applications is a transdisciplinary field that in recent years has involved researchers from chemistry, pharmacy, medicine, biology, biophysics, and biomechanical engineering. The fabrication of biomedical devices requires the use of biocompatible materials that do not damage living tissues and have some biomechanical characteristics. The use of polymeric membranes, as materials meeting the above-mentioned requirements, has become increasingly popular in recent years, with outstanding results in tissue engineering, for regeneration and replenishment of tissues constituting internal organs, in wound healing dressings, and in the realization of systems for diagnosis and therapy, through the controlled release of active substances. The biomedical application of hydrogel membranes has had little uptake in the past due to the toxicity of cross-linking agents and to the existing limitations regarding gelation under physiological conditions, but now it is proving to be a very promising field This review presents the important technological innovations that the use of membrane hydrogels has promoted, enabling the resolution of recurrent clinical problems, such as post-transplant rejection crises, haemorrhagic crises due to the adhesion of proteins, bacteria, and platelets on biomedical devices in contact with blood, and poor compliance of patients undergoing long-term drug therapies.

## 1. Introduction

Membranes are defined as films that can work as a separation barrier between two adjacent phases, and due to their porosity, they allow the selective passage of substances from one phase to the other one [1,2]. Polymer membranes can have a porous structure, in which separation is based on pore size differences, or a dense structure [3], in which separation is based on the solubility and diffusivity of molecules. Permeability, selectivity and flux are the most important characteristics of polymeric membranes. Their classification takes place according to several criteria: nature of the polymeric material (natural or synthetic); structure (symmetrical or asymmetrical, porous or nonporous); mechanism by which separation is achieved (the size of the permeant species and their solubility in the membrane); and physical chemical properties of the membrane (hydrophobic or hydrophilic).

If the classification of membranes is made according to their nature, they can be divided into natural and synthetic membranes. The latter can be further classified, according to material, into organic, inorganic, or composite membranes [4]. Organic membranes are by far the most widely used. They are made of synthetic polymers such as poly(ethylene glycol) (PEG), poly(acrylic amide), polysulfone, polyurethane, poly(N-vinyl-2-pyrrolidone), or they can be hydrogels based on natural polymers such as hyaluronic acid, cellulose, chitosan, alginate, collagen and others [5,6,7,8]. Membranes can also be classified according to pore size as porous, dense, and asymmetric [9]. In porous membranes polymers usually occupy only a small part of the total volume. Depending on the pore size, these materials can be further subdivided into microporous, if the pore diameter is less than 10 µm, and macroporous if the pore diameter exceeds 10 µm.

Dense membranes have no real pores but still have voids formed by the spaces between molecular chains (the so-called “free volume”) of the order of 5–10 Å.

Asymmetric membranes have a dense and very thin outer film (skin) (0.1–0.5 µm thick), which is responsible for the membrane’s selective behavior, and a thicker porous support (0.1–0.2 mm). The presence of the outer film enables simultaneous selectivity and high fluxes, while the porous support is responsible for mechanical properties and facilitates handling.

The use of membranes has become increasingly established in the biomedical field, and particularly in tissue engineering, with the realization of biological purification systems, protective coatings for wounds that accelerate healing, systems for diagnosis and therapy through the controlled release of active substances [10], and scaffolds. Scaffolds are the main resource of tissue engineering, which is concerned with restoring the functionality of damaged tissues and organs. They are temporary or support artificial structures with nanometric morphological characteristics engineered to emulate the extracellular matrix (ECM) in order to house and support cell cultures and promote their growth until regeneration of the damaged tissue is achieved.

The development of biomedical materials requires that they exhibit particularly high levels of biocompatibility, bioactivity, and biofunctionality [11,12,13,14,15,16,17]. Research has pursued to obtain attractive material properties, including biomimetic methods, inspired by the principle that living organisms within their structures exhibit well-tested strategies [18,19,20,21].

Cells are the basic structures of living organisms. Cell membranes are a nanostructured molecular assembly consisting of lipids, glycolipids, transmembrane proteins and peripheral proteins. The many elements of cell membranes, such as chemical constituents, structure, membrane channels, and receptors can inspire the design of biomaterials, or these elements can often constitute interaction sites for biomedical polymers [22,23,24].

Among the polymers that have recentry successfully employed for biomedical applications, hydrogels have assumed a relevant role. A hydrogel is a material consisting of hydrophilic polymers capable of swelling water and retaining it in amounts that can reach up to 1000 times their dry weight. The three-dimensional structure that such polymers form due to the cross-linking of the individual polymer chains has characteristics of great flexibility very similar to that of natural tissues. Hydrogels can be prepared with two types of cross-linking: hydrogels with covalent cross-linking are called chemical gels, while hydrogels with noncovalent interactions are called physical gels [25]. On the cross-linking density depends the control of the porosity of the hydrogel and thus also the ability to load the drug inside it.

Hydrogel membranes combine the porous architecture and permeability properties of thin membranes with the dynamic mechanical properties and water absorption characteristics of polymeric hydrogels. The characteristics of hydrogel membranes bear a remarkable resemblance to physiological membranes, although the latter are much more complex than their synthetic counterparts. In any case, such characteristics make hydrogel films promising to produce membranes for various applications [26,27], such as antimicrobial coatings, cell culture substrates, wound dressing applications, scaffold production [5], artificial tissues engineering, drug delivery systems.

**Figure 1:**
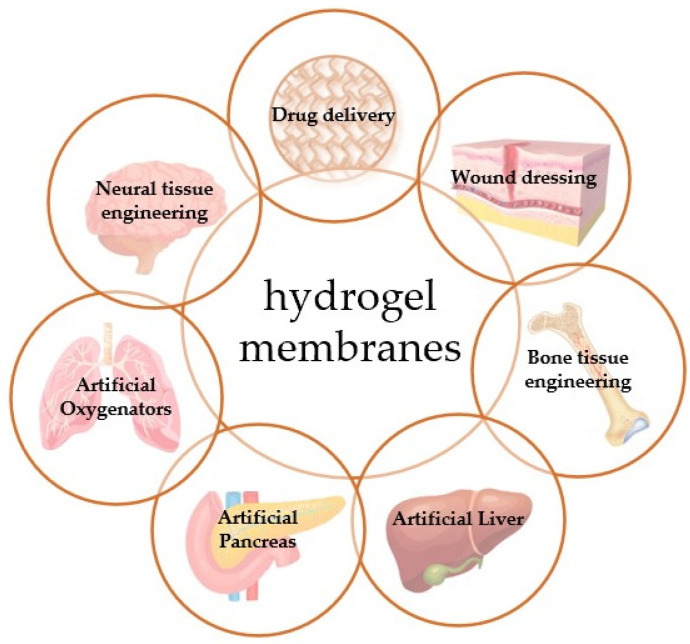
Hydrogel membranes and the biomedical fields in which they are being exploited.

This review deals with hydrogel membranes applications in various biomedical fields (Figure 1). The results obtained in many works concerning this argument are commented.

## 2. Liver

Chronic liver diseases (CLD) are among the most life-threatening diseases in humans, and have causes that can be traced to obesity, non-alcoholic steatosis, high alcohol consumption, hepatitis B or C infection, autoimmune diseases, cholestatic diseases, and iron or copper overload [28]. Such diseases can give rise to chronic inflammation of the liver and cause a decreased liver function, which can result in the development of cirrhosis.

The obvious inconveniences in approaching the surgical solution of transplantation have suggested researchers develop an artificial liver capable of performing the same functions performed by the liver, namely purification, excretion and biotransformation [29].

Research groups have developed techniques that allow the in vitro production of primary human hepatocytes [30,31].

Numerous publications testify to the use of hydrogels as supporting biomaterials regenerative medicine [32,33,34,35,36,37].

However, there is no well-defined protocol for the use of different hydrogels in the field of liver tissue engineering (LTE). In fact, to date, no hydrogel that mimics liver extracellular matrix (ECM) cells are available. Therefore, the use of LTE is limited to the creation of in vivo models [38,39].

**Figure 2:**
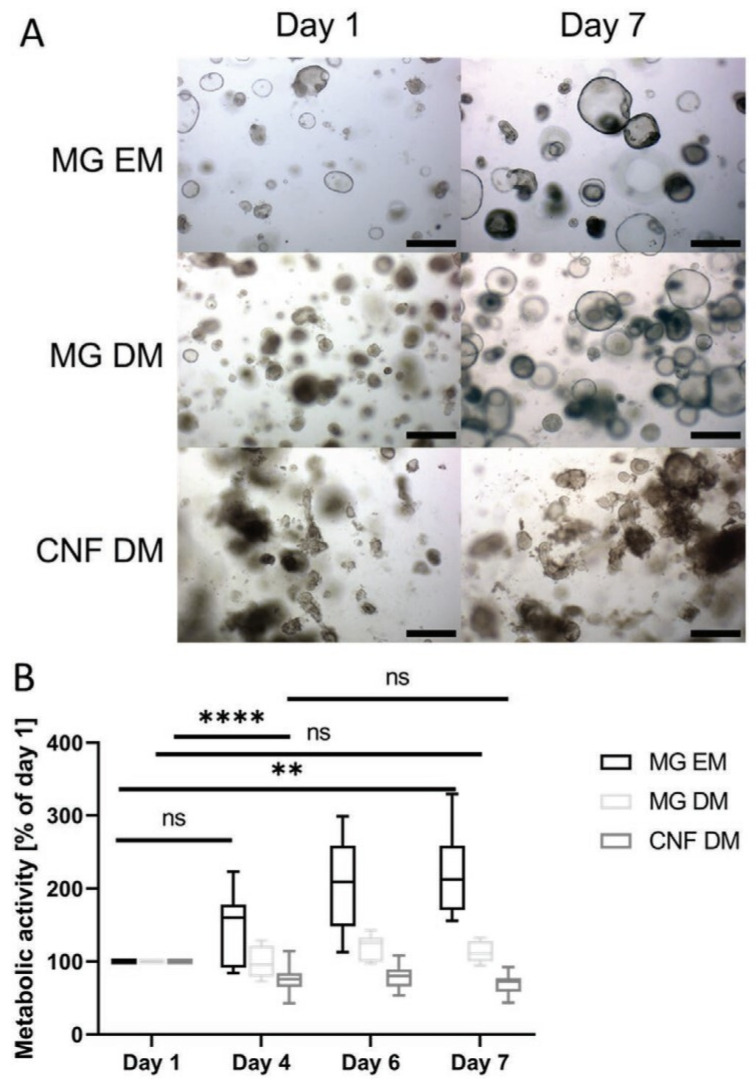
(**A**) Representative brightfield images showing liver organoid morphology on days 1 and 7 after transfer into Matrigel (MG) under expansion medium (EM) or differentiation medium (DM) conditions, or in cellulose nanofibril (CNF) hydrogel 0.4% (*w*/*v*) under DM conditions, scale bar 500 μm. (**B**) Metabolic activity at days 4, 6, and 7 relative to values at day 1 (*n* = 9 for MG EM and DM, *n* = 24 for CNF DM); data depicted as box with median and 25th and 75th percentile and whiskers from min to max (** *p* < 0.01, **** *p* < 0.0001, ns: not significant), ANOVA with Tukey’s post-hoc test [40] (License CC BY).

Among natural polymers, cellulose could fulfil, in combination with human liver organoids, many characteristics that could ensure successful liver tissue engineering. Liver organoids derived from adult liver have been found to be very attractive because of their genetic stability and ability to differentiate to hepatocyte-like potential. Until recently, basement membrane hydrogels were used to culture these organoids, among them Matrigel (MG), derived from murine tumour material. It, possessing an indefinite composition and particularly high cost, is not applicable in the clinical setting. Kruger et al. [40] studied a hydrogel based on cellulose nanofibrils (CNFs) with the aim of making an alternative method for hepatic organoid differentiation. The results showed mechanical properties, such as self-repair and shear thinning behaviour, suitable to induce organoids’ differentiation ability, equal or even improved compared to that induced by MG, as can be seen in Figure 2.

Therefore, CNF hydrogel presents as a viable alternative to MG for liver tissue engineering, with the possibility of being employed for clinical use. Improvements could be achieved by producing smaller CNF microgels, further adjusting the elastic modulus of the hydrogel, introducing cellulase enzymes [40], or even bioactive groups to the nanofibrils.

In recent years, decellularized ECM-based hydrogel materials have attracted attention for their excellent biocompatibility [41,42]. Some studies have reported that decellularized liver matrix coating on 3D cryogels can promote hepatocyte growth and function [43]. As reported by Asadi et al. [44], synthetic hydrogels produced by the combination of decellularized ECM (dECM) and Poly(N-isopropylacrylamide) pNIPAAm (dECM-pN) were found to preserve intact hepatocyte function in cell sheets, and the ECM synthesized from the hydrogels could reconstruct the microenvironment to induce adhesion, proliferation, and differentiation of Adipose Tissue-derived Mesenchymal Stromal Cells AT-MSCs.

Recently, researchers have treated mouse liver after decellularization to form hepatic hydrogels. Experiments have also been conducted to show that hepatic hydrogels can reduce liver fibrosis by replacing necrotic hepatocytes and damaged ECM [45].

**Figure 3:**
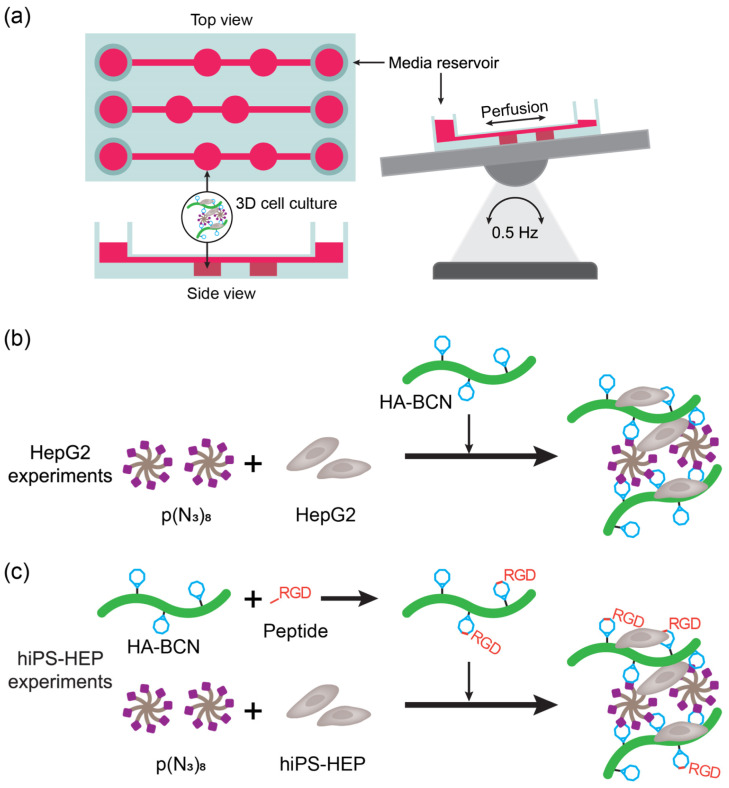
(**a**) Schematic representation of the liver-on-a-chip device and setup. The device was put on an automatic rocker table to allow perfusion of media and nutrients during cell culture. (**b**) Depiction of the liver carcinoma cells HepG2 3D cell culture experiments with hyaluronan-poly (ethylene glycol) (HA-PEG) hydrogels. The HepG2 cells were added to media-suspended p(N_3_)_8_ ((8-Arm PEG-Azide) prior addition of cyclooctyne-modified hyaluronan (HA-BCN). (**c**) Depiction of the pluripotent stem cell derived hepatocytes (hiPS-HEPs) 3D cell culture experiments with HA-PEG hydrogels. The hiPS-HEP cells were added to media-suspended p(N_3_)_8_ prior addition of HA-BCN. In experiments using either linRGD or cRGD peptide, the HA-BCN was preincubated with 1 μM of corresponding peptide for 1 h prior adding the HA-BCN(RGD) component to the hiPS-HEP/p(N3)8 mixture [46] (License CC BY).

In conventional in vitro models, the use of two-dimensional (2D) surfaces for cell seeding can lead to altered cell function [46]. To better mimic the in vivo cell environment [47,48], recently miniaturized bioreactors, in which cells come to be spatially arranged and cultured under dynamic conditions, have been proposed as alternative cell culture models and have become popular as “organs-on-a-chip”. One approach to enhance and prolong cellular functions within organs-on-a-chip is to encapsulate cells within a three-dimensional (3D) matrix of hydrogels that mimics the supporting functions of the extracellular matrix (ECM) [46,49]. Specifically in ref. [46] Christoffersson et al. highlighted the advantages of using a hyaluronan-PEG-based hydrogel modified with RGD peptides to achieve hepatocyte culture within a liver-on-a-chip system. To adjust the biofunctional viscoelastic properties the production of the hydrogel was by performing a copper-free click chemistry approach. The encapsulated liver carcinoma cells HepG2 cells-maintained viability and functionality for more than 9 days. the pluripotent stem cell derived hepatocytes (hiPS-HEPs) Figure 3 illustrates the procedure to set up the liver-on-a-chip.

## 3. Pancreas Regeneration

**Figure 4:**
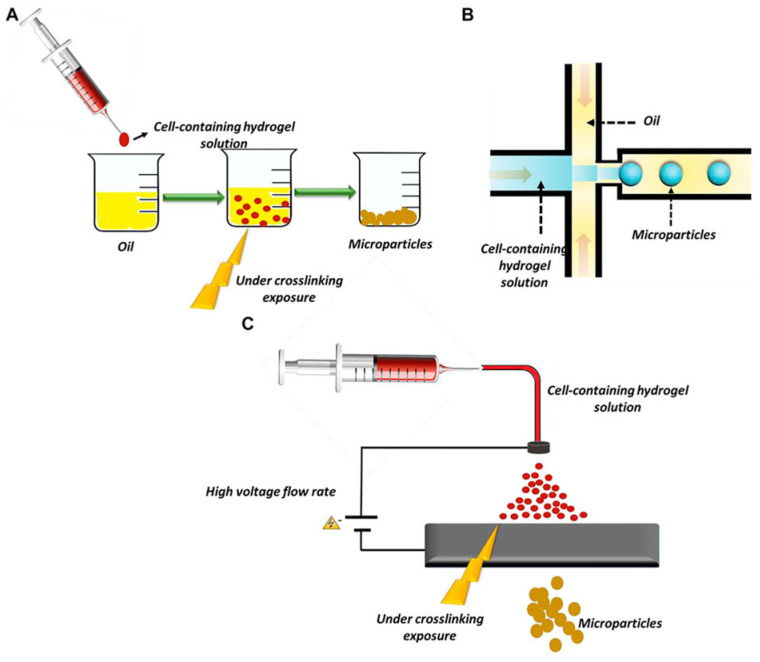
Methods of microparticles fabrication: (**A**) batch emulsion technique, (**B**) microfluidic emulsion technique, and (**C**) EHD spraying [53] (License CC BY).

Type 1 diabetes mellitus (T1DM) is a chronic metabolic disease characterized by the insufficient or absent production of insulin, due to β-cell destruction [50], which can result in hyperglycaemia. According to the World Health Organization (WHO), diabetes is one of the ten leading cause of death and has a large incidence in risk of heart attacks, strokes, cardiovascular disease, kidney failure, and blindness. The conventional therapeutic approach for treating T1DM is insulin replacement therapy, which requires continuous subcutaneous injections of insulin. Such therapy effectively controls blood glucose levels but is unable to recapitulate physiological pancreatic insulin, and thus cannot completely curb the possibility of developing the diseases listed above. Pancreatic islet transplantation is an alternative and less invasive therapeutic method. Unfortunately, the rejection of the transplant by the immune system and the lack of islet donors do not allow for widespread use of transplant protocol [51]. The limitations of this approach can be made less important by protecting the transplanted secretory cells against the recipient’s immune system, allowing the transfer of insulin, oxygen, and other nutrients [52]. Microencapsulation, intravascular or extravascular, depending on the location of the implant, of β-cells, using biomaterials, has been proposed. Among the various methods spreadly used in β-cell encapsulation (Figure 4) batch emulations, microfluidic emulsions and EHD spraying must be mentioned [53].

Several studies have considered hydrogels as a material to be exploited for the encapsulation of islets. Haque et al. [54] used injectable Matrigel^®^, a thermosensitive hydrogel membrane derived from ECM, also used for liver organoid expansion [40], as a carrier to inject subcutaneous β-cells together with liposomal clodronate, a novel agent that could improve islet survival. Results showed that this cell delivery system increased islet survival from 10 to 60 days [53].

The chemical and physical characteristics of the natural polysaccharide alginate, which is biocompatible and inexpensive, make it a biomaterial with suitable prerequisites for the fabrication of hydrogel membranes that can be used for β-cell encapsulation. In a work published in 2018 [55], the preparation of an injectable alginate-based hydrogel was described. This preparation was done by ion cross-linking, and retarding, using Na_2_HPO_4_, the gelation time. Lengthening the gelation time of the hydrogel imparted mechanical properties, that were particularly useful in clinical applications, such as manageability and flexibility, but also physiological properties that improved biocompatibility and β-cell growth.

**Figure 5:**
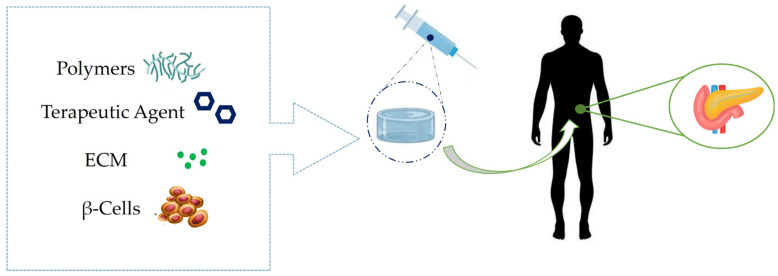
Synthetic scheme of membrane hydrogels for pancreatic therapy. On the left various components of a generic microencapsulation systems for β-Cells are shown.

Wang et al. [56] produced interpenetrating thermosensitive networks (IPNs) based on alginate and ECM derived from human adipose tissue, to encapsulate β islets (Figure 5). The procedure was conducted by introducing the islets into an alginate solution and then performing cross-linking by ionic gelation. This structure was added to the hydrogel of the ECM. In vitro cell studies have found the system to be biocompatible and not susceptible to immune attack. Within the time span of one week, there was a significant (seven-fold) increase in cell population compared with when the cells are not encapsulatedSynthetic PEG-based hydrogels, due to their low protein adsorption, minimal inflammatory aggressiveness, immunoprotective properties, and high biocompatibility, are promising vectors for cell delivery [57]. In a study Knobeloch et al. [58] developed an injectable PEG hydrogel that supports islet survival in vitro and in vivo The hydrogel was produced by subjecting a multiarm of PEG-vinyl sulfone (VS) and PEG dithiol as the cross linker to reaction (Michael’s addition). The islets were encapsulated in the hydrogel and the solution was injected about 90 s before gelation. Determination of blood glucose levels showed a significant reduction from 600 to 200 mg/mL as early as about 2 days after implantation.

## 4. Artificial Oxygenators

Recently, due to the COVID-19 pandemic, the need for the development of artificial oxygenators has grown. Oxygenators are defined as medical devices designed to provide breathing support. Among those, most widely used in the treatment of critically ill patients with cardiopulmonary compromise caused by infection [59,60,61], is extracorporeal membrane oxygenation (ECMO). The operation of oxygenators is based on this principle: when blood passes through the oxygenator, the oxygen level increases and the CO_2_ level decreases, to oxygenate the non-oxygenated blood [62,63,64]. Extracorporeal membrane oxygenators consist of a pump that has the role of pumping the blood and an oxygenating membrane that has the role of oxygenating the blood. The most common causes of complications in patients using an oxygenator are clotting and bleeding [59]. The material from which the oxygenator membrane is obtained must have high permeability, high mechanical strength, no defects, high biocompatibility and hemocompatibility [65]. Adhesion of proteins, bacteria and platelets on biomedical devices in contact with blood is the main cause of thrombosis and infection [66]. To prevent clot formation in the oxygenation system, anticoagulant drugs, such as heparin, are administered. Unfortunately, this method is not without serious risks, such as those of haemorrhage [67,68].

Functional coatings [69] play an important role in the emerging field of medical devices, helping to achieve appropriate molecular interactions that can curb problems inherent in the activity for whose purpose the membrane was designed. Numerous studies have shown that zwitterionic polymers are ideal candidates for preventing thrombosis and infection formation due to their superhydrophilic properties through equal amounts of positively and negatively charged groups on the same side chain [70,71]. In general, the formation of zwitterionic polymer brushes on biomedical devices in contact with blood is a promising strategy. Hydrogel coatings show even more promise due to characteristics such as thickness, lubricity, and hydrophilicity, which can be adjusted [72]. However, limitations to the application of zwitterionic hydrogel coatings in anti-thrombosis and anti-infection medical devices arise from the superhydrophilic characteristics [73], which weaken their mechanical properties, while it is important that the coatings have robust mechanical properties and strong device adhesion [74]. To improve mechanical properties, non-zwitterionic components are usually introduced. Yao et al. [75] created a zwitterionic hydrogel coating reinforced with poly(carboxybetaine) (pCBM) microgel. The microgel was first prepared by reverse polymerization in miniemulsion, and then combined with poly(-sulfobetaine) (pSB). In this system, the pSB, as a continuous phase, passes through the pCBM (which acted as a crosslinker), to increase the mechanical strength. The pCBM/pSB hydrogel was used as a coating of polyvinyl chloride (PVC) pipes. Detailed studies of the mechanical and stability properties of the pCBM/pSB hydrogel coating were carried out. In addition, the antithrombogenic properties were investigated by making an extracorporeal circuit (ECC) of SD rats and New Zealand white rabbits.

An anticoagulant coating consisting of a methacrylate alginate hydrogel (MA-SA) was synthesized using a UV cross-linking reaction and then applied to PVC pipes [76]. Natural polysaccharides exhibit similar anticoagulation mechanisms to heparin, and thus can replace it as a viable alternative. Sodium alginate, through its sulfated polysaccharide site, can bind to antithrombin III (AT-III), catalyzing its action and antagonizing coagulation factors IIa, Xa, IXa, XIa and XIIa. Thus, the intrinsic coagulation pathway, which involves the conversion of prothrombin to (IIa), and the activity of thrombin come to be inhibited; in addition, the conversion of fibrinogen to fibrin monomer is hindered.

**Figure 6:**
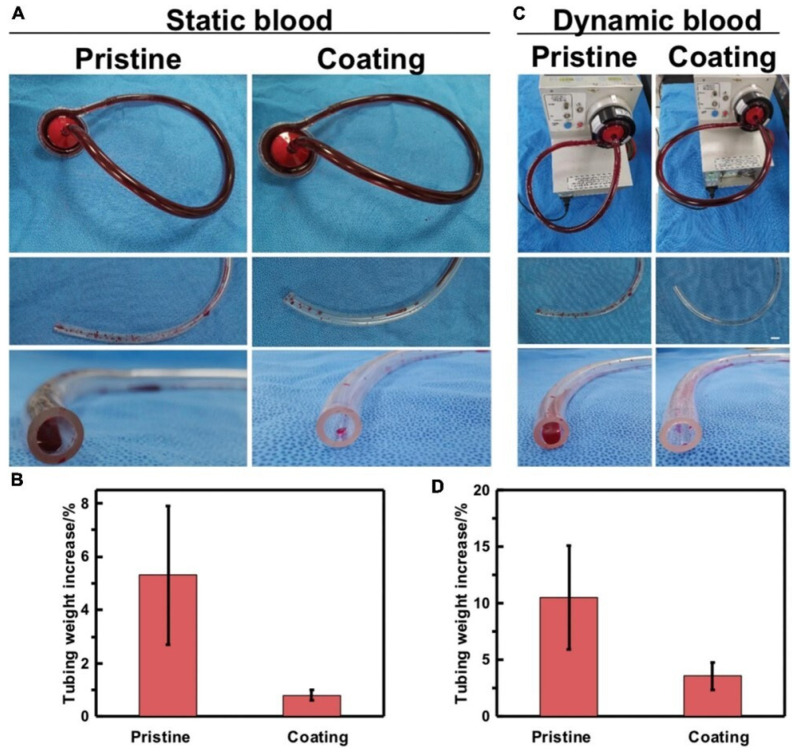
In vitro blood loop tests of static blood and dynamic blood. (**A**) Image of apparatus used for continuous flow testing of pristine and coated tubing. Testing was done in parallel to accurately control the end time point of flow. (**B**) Quantification of blood clotting adhesion to the tubing walls in static blood. (**C**) Image of pristine and coated tubing after flow testing and gentle rinsing with saline. (**D**) Quantification of blood clotting adhesion to the tubing walls in dynamic blood. Error bars represent SD for three repeated experiments [76] (License CC BY).

The surface of MA-SA-hydrogel coatings was irradiated with UV light to polymerize the hydrogel layer. The obtained MA-SA hydrogel coating was found to be uniform and transparent. The morphology of the ECMO surface coated with MA-SA-hydrogel has a porosity of about 5 mm, however, the diameter of RBCs observed under the light microscope is less than 8 mm [77]. The contact angle is 112°, which proves the hydrophobicity of MA-SA-hydrogel coating, that is a low inclination to thrombosis. Blood was circulated through the circuit depicted in Figure 6. During the dynamic blood experiment, if the tube is not coated, there is an average weight gain of 10.5 percent, while the coated tube in Figure 6D shows a weight gain of 3.6 percent. This suggests that blood clots adhered more readily to the uncoated tube than to the MA-SA-coated tube. All experiments showed that modification of the PVC surface with the hydrogel coating of SA leads to platelet activation, thrombosis, and blood incompatibility. In contrast, M-SA hydrogel coatings decreased platelet adhesion and activation, thereby improving anticoagulant performance. MA-SA hydrogel coating technology, therefore, may be a useful procedure to curb the risk of thromboembolism associated with the use of blood-contact medical devices.

## 5. Wound Healing

Wound healing is a process that involves several interdependent steps, in which the activity of cellular components that promote tissue regeneration and growth takes place [78,79]. The overall process is divided into homeostasis, inflammation, proliferation, and re-epithelialization [80,81]. Infections at the hands of microorganisms, that can take over when wounds are left untreated, adversely affect the healing process by lengthening its time.

In order to find a versatile wound dressing, different types of materials such as membranes, hydrogels, films, foams, microparticles, and nanoparticles have been used [82,83].

**Figure 7:**
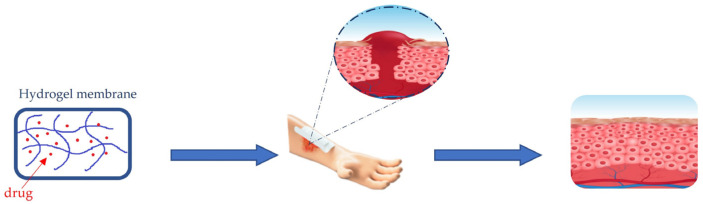
Healing process of wounds trough hydrogel membrane dressing.

Among the most used materials for acute and chronic wound management are hydrogels. Hydrogel membranes act as a moist dressing [84]. The release of moisture increases collagenase production and provides a suitable environment for tissue regeneration (Figure 7).

The cross-linked structure of polymer chains in hydrogels can expand as a gel mass, absorbs and retains exudates, isolates bacteria, odorous molecules and debris from the exudate. The high aqueous content helps diffuse oxygen and vapor into the wound, thus providing a soothing effect.

Abbasi et al. [85] synthesized heat-sensitive hydrogel membranes, using sodium alginate biopolymer, synthetic polymer F127 and PVA as the cross-linker. The thermosensitive hydrogel showed good mechanical properties, elasticity, flexibility and tensile properties related to the degree of polymer crosslinking. Porosity on the polymer surface allowed ‘oxygenation of the wound, contributing to a stimulating environment for its re-epithelialization. The porosity allowed sustained release of the antimicrobial drug, which promoted accelerated healing. The drug loaded was amikacin, which has strong activity against gram-positive (*S. aureus*) and gram-negative (*P. aeruginosa*) organisms. Histological examination performed on an animal model attested the complete wound healing in 21 days.

Batool et al. [86] made a PVA/Starch-based membrane hydrogel in which silver nanoparticles (NPs) were embedded. These NPs were extracted from the *Diospyros lotus* plant through two different methods, one green and one nongreen. The former used water and the latter used methanol as a solvent.

Nanoparticles, in general, have several advantages, such as greater stability, longer shelf life and pharmacological effect of the drug, which in turn increases bioavailability and reduces dosing frequency [87]. Nanomaterials can promote wound healing through direct regulation of the extracellular matrix, promoting stem cell growth and skin regeneration by modulating growth factors at the wound site. Due to the unique properties of high surface-to-volume ratio, nanoscale size and porosity, they are used in wound dressings care and management [87,88]. The silver NPs obtained by the two different methods were studied and showed very similar properties between them. Next, the mechanical and also antibacterial properties of the membranes with and without the NPs were compared. The mechanical properties of membranes without NPs were found to be better, while NP membranes showed superior swelling and moisture retention capabilities. Membranes that had incorporated NP prepared with organic extract also exhibited antibacterial activity, which was totally absent in membranes without NP. Membranes containing “green” Ag NP thus have great aptitude for use in wound dressing applications.

Among the natural polymers most widely used in the manufacture of hydrogel membranes, there are hyaluronic acid and chitosan. Hyaluronic acid is a non-sulfur anionic glycosaminoglycan [89], which often occurs in the form of a sodium salt. It exhibits unique characteristics, including biocompatibility, biodegradability, nonimmunogenicity, and hydrophilicity [90].

Chitosan is a linear cationic polyamide, it is obtained by deacetylation of chitin, the second most abundant biopolymer in nature, after cellulose. It has bactericidal and bacteriostatic action. It is biocompatible and has low toxicity in wound dressings. It provides a moist environment to heal wounds, prevents the accumulation of exudates and reduces the chances of bacterial infection [91].

Shafique et al. [92] used hyaluronic acid and chitosan to produce membranes consisting not only of these two natural polymers, but also of pullulane and polyvinyl alcohol (PVA). Pullulane is a polysaccharide polymer composed of maltotriose units connected by α 1–6 bonds [93]. It possesses great advantages such as low toxicity and mutagenicity, high biodegradability, and water solubility. Above all, pullulane can form thin films with structural flexibility, adhesive properties and great mechanical strength [94,95]. PVA is a hydrophilic polymer, which can retain water, providing a moist environment and thus conducive to wound healing. It is biocompatible and biodegradable with good mechanical properties. The antimicrobial properties of the hydrogel membrane, due to the presence of chitosan, have been enhanced through loading with nanoparticles of cefepime, an antibiotic belonging to the fourth generation cephalosporins, which is usually parenterally administered. It is effective against Gram-positive pathogens such as MRSA, PRSP, *Streptococcus pyogenes*, and Gram-negative pathogens such as *Escherichia coli*, *Klebsiella pneumonia* and *Serratia*, *Citrobacter* [96,97]. The membrane hydrogel, tested on an excisional rat model that showed rapid recovery, demonstrated inhibitory action especially against the proliferation of *Staphylococcus aureus*, *Pseudomonas aeruginosa* and *Escherichia coli.* The important antibacterial activity seems likely to warrant promising use by skin application of the produced membrane as a potential accelerator in the wound healing process.

During wound healing, if a disproportionate inflammatory response occurs, the resulting increase in wound size complicates the process of tissue regeneration. (S)-ibuprofen (IBP), a nonsteroidal anti-inflammatory agent used for healing muscle injuries and treating venous leg ulcers, has also been studied as an active ingredient for skin wound healing. Agujar-Ricardo et al. [98] designed IBP-β-cyclodextrin carriers to modulate the release of IBP from poly (vinyl alcohol)/chitosan (PVA/CS) dressings with the aim of achieving faster skin regeneration. In vitro studies showed that β-cyclodextrins allowed controlled release of IBP from hydrogels, while in vivo assays revealed that the presence of PVA/CS membranes prevented crusting and excessive inflammation, accelerating the healing.

## 6. Bone Tissue Engineering

**Figure 8:**
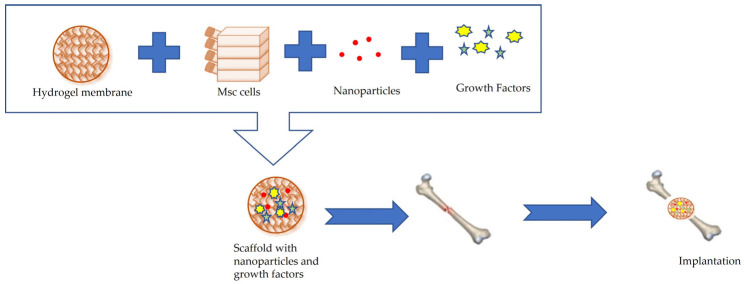
Bone tissue regeneration through the use of hydrogel membranes supplemented with inorganic nanoparticles The system can promote the differentiation of bone Marrow Stromal Cells (Msc) and can be integrated in the damaged bone.

Bone tissue is made up of various types of cells, and its ECM contains both organic elements, such as type I collagen, and inorganic elements, such as hydroxyapatite [99]. Inorganic substances can give bone a special hardness. Research in recent years has developed many membranes to accelerate bone regeneration after traumatic events resulting in injury. Hydrogels have been used in various therapeutic treatments (Figure 8), such as, for example, for filling bone gaps [100,101].

A multilayer hydrogel membrane consisting of chemically converted graphene (CCG) has been used as a barrier membrane for bone regeneration [102]. In a rat model both osteoinductivity and osteoconductivity were increased, which resulted in improved mineralization of mature lamellar bone. This was interpreted as an effect of the osteogenic activity of CCG and multilayer membrane nanostructure.

Fracture healing is a process that takes place in several stages [103]. There are factors that can hinder this process: soft tissue damage, location of the injury, age of the patient, osteoporosis, and use of particular drugs. In orthopaedic and joint/prosthetic surgery, infections are a not uncommon complication [104,105].

Johnson et al. [106] designed injectable hydrogels to treat infections caused by *Staphylococcus aureus* in orthopaedic implants used for fracture repair. A mouse model of femoral fracture infection was used to evaluate the therapeutic potential of lysostaphin therapy incorporated into a formulation consisting essentially of a PEG hydrogel. By adhering to exposed fracture surfaces, the formulation allowed lysostaphin to be effectively administered locally. Lysostaphin encapsulated in this synthetic hydrogel maintained its stability. The released lysostaphin showed greater antibiofilm activity than the unencapsulated lysostaphin. Thus, the authors demonstrated that PEG-based hydrogels can restore the fracture healing process, which has been altered by infection sustained by *Staphylococcus aureus*. In addition, hydrogels can deliver growth factors added directly to the gel to promote fracture healing.

The main constituents of osteochondral tissue are subchondral bone and articular cartilage. To correct defects in this tissue, regeneration of both articular cartilage and subchondral bone is necessary [107,108].

Due to their characteristics of biocompatibility, biodegradability, and control of cell-ECM interactions, hydrogels have emerged as a material of choice for the fabrication of membranes suitable for cartilage tissue repair [109].

In recent years, great strides have been made in the field of cartilage tissue engineering, such as using 3D printing and doping of hydrogels with porous and/or biodegradable microspheres to induce cartilage structure formation. The porosity plays an important role. This is demonstrated by the fact that in scaffolds with closed pores, cells are poorly distributed, thus generating an inhomogeneous ECM, characterized by poor mechanical properties. Hydrogels are being used as a basic biomaterial for cartilage recovery through two modes: the first involving a carrier action of cells that go on to promote tissue regeneration, and the second as a constituent of permanent implants for the replacement of damaged cartilage tissue [109]. The polymers most commonly used as base material of hydrogels are Polyethylene glycol diacrylate (PEGDA), hyaluronic acid thiolates, chitosan, graphene, and alginate.

Zhu et al. [110] combined 3D-printed acellular chondrocytes, extracellular matrix (ECM), polyethylene glycol diacrylate (PEGDA), and Honokiol, a natural compound that revealed good anti-inflammatory properties for the treatment of various diseases, inclu-ding osteoarthritis. The combination tested showed promising results for the recovery of osteochondrial defects.

Yuan et al. [111] prepared composite material of HAPNW HydroxyAPatite NanoWires embedded in a double network of bovine serum albumin/sodium alginate. HAPNWs were added to the hydrogel membranes not only to improve their microstructure, but also to increase their mechanical properties. In fact, the resulting material possesses higher porosity, swelling and compressive modulus properties. In addition, in vivo studies have confirmed that the obtained material can increase the proliferation and differentiation of bone marrow stromal cells (BMSCs) and promote the integration of the regenerated tissue with the surrounding normal tissue.

## 7. Neural Tissue Engineering

Neurological diseases can be severe and difficult to treat. Neural tissue engineering offers valuable help through the selection of basic materials to produce suitable membranes to promote neural cell differentiation and growth [112]. Hydrogels are among these materials. They have been exploited for the delivery of neural growth-promoting agents and neurotrophic factors that oppose neural growth inhibitors (chondroitin sulphate proteoglycans (CSPG) [113], Nogo [114], and myelin-associated glycoprotein [115]. Recently encapsulating hydrogels have been used to protect neural cells from immune activity.

The term “neural tissue” seems to refer mainly to neurons. Actually, neural tissue engineering is aimed at developing functional neural tissue not only of neurons but also of non-neuronal glial cells [116].

An important element, which influences attachment, the creation of neuronal synapses and the regulation of their diameter, is the maintenance of mechanical tension along the neurite. It also influences the arborized arrangement of neurons [117]. In synthetic and natural hydrogels such as those of polyacrylamide and fibrin, it has been observed that cell survival and neuritic extension of cortical neurons cultured on them are higher when the elastic modulus of the hydrogel is closer to that of the extracellular matrix [118]. Softer gels increase neuronal sprouting to a greater extent than harder gels. In contrast, astrocytes develop much better on stiffer substrates.

Several research papers in the last two decades have reported [119] that electrical stimulation of damaged neural tissue can give important contributions to its repair and regeneration. The mechanism underlying electrical stimulation has not been fully elucidated, but several hypotheses have been made, including those regarding the role of voltage-dependent calcium channels [120] and changes in the local electric field of extracellular matrix molecules [121].

Lee et al. [122] developed a material based on a PEG hydrogel substrate micro patinated with a silver nanowire (AgNW). The introduction of silver NWs increased the conductivity of the substrate, which was sensitive to electrical stimuli applied to differentiate neural stem cells (NSCs) and to drive the growth of neurites. To further guide the growth of neurites, parallel micro arrays were created from the hybrid hydrogel material. The combination of electrical stimuli and physical micropatterns containing AgNWs in one device resulted in synergistic effects, with a neurite outgrowth rate higher than that obtained using electrical stimuli or micropatterns alone.

Liu et al. [123] produced a perfluoro polyether thin film functionalized with dimethacrylate and subjected to crosslinking by UV. It can perform localized neuromodulation by interfacing with peripheral nerves. The Young’s modulus of this material was adjusted to match that of nerve tissue. The authors then described the lithographic process developed to pattern the soft and intrinsically stretchable material in a multi-electrode array. The results of the study validated the biocompatibility and the stability of the system in an aqueous environment. It was able to perform good electrical stimulation with ultra-low voltages.

**Figure 9:**
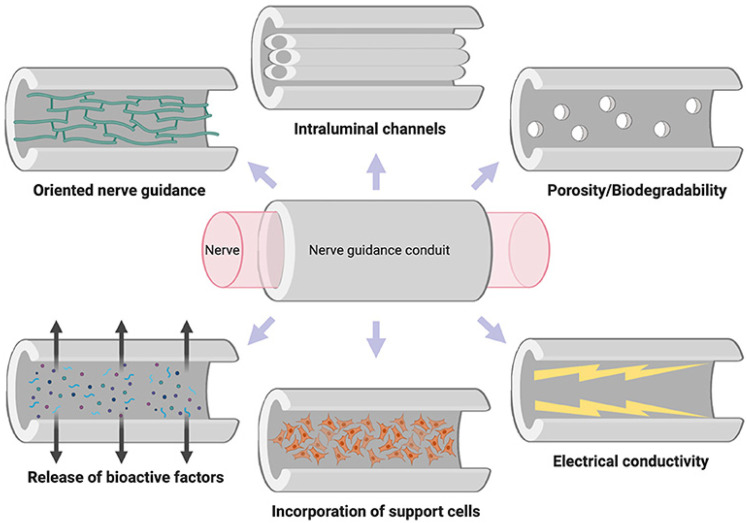
Properties of the ideal nerve guidance conduit [124] (License CC BY).

For the future, we can say that hydrogel membranes are presented with ideal properties for neuronal tissue growth and regeneration, and may supply a platform that can provide, separately or synergistically, cues for the replacement and the repair of neural components, with restoration of function (Figure 9) [124].

## 8. Drug Delivery

Ideal drug delivery must ensure efficiency and avoid side effects. From this it follows that the drug concentration at the plasma level must be effective and below the level of toxicity as far as possible [125]. The traditional method of drug administration generally results in the plasma concentration rising to a peak and then decreasing. This leads to an inevitable risk of toxicity and drug wastage. One solution could be smart membranes, which can ensure stable and targeted drug release, plus incorporating features responsive to environmental stimuli into drug delivery systems [126,127]. For controlled drug release, smart membranes can be made in the form of capsules, which are easy to design and apply. Such stimuli-reactive capsule membranes, with a shell structure, can provide a large internal volume for encapsulation of various drugs and in a versatile manner for controlled release [128].

**Figure 10:**
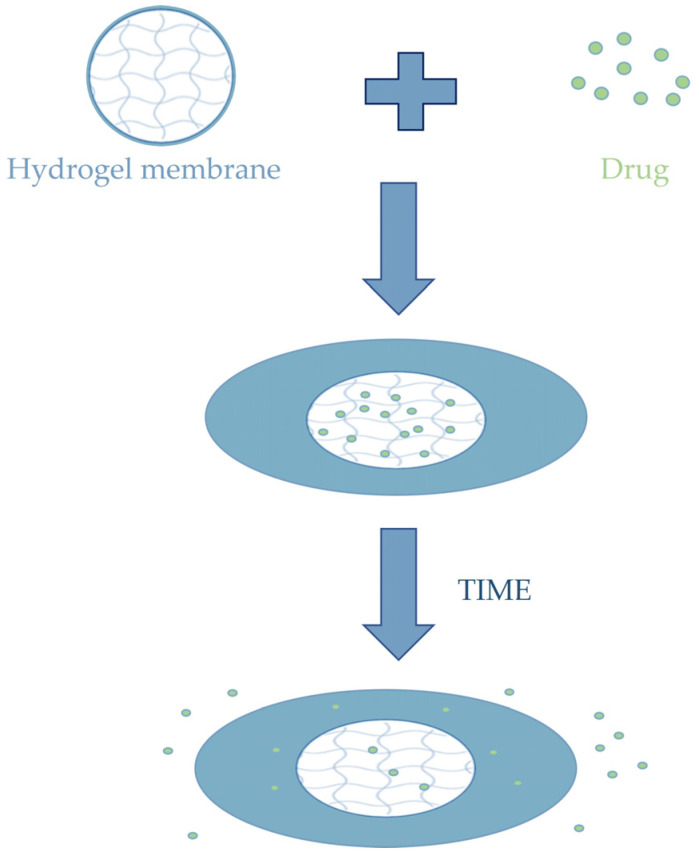
Loading and releasing of a drug by a hydrogel membrane.

Hydrogels, due to their three-dimensional polymer structure and their ability to absorb a large fraction of water, can be exploited for controlled drug release (Figure 10). Control can be accomplished through special stimuli such as changes in pH, temperature, and electrical potentials. What is particularly interesting to researchers is the possibility of making drug release very precise and timed [129,130]. That is why scientists have increasingly turned toward the study of smart systems that can pick up signals produced by the disease and release the specific amount of drug responsive to the physiological condition, minimizing the risk of side effects. To achieve efficient controlled release and low side effects, an on/off model seems to offer the greatest assurance. It is ideal for the treatment of chronic diseases that require frequent administration. For example, for blood glucose level control, subcutaneous insulin injections are given in diabetes, an inconvenient and painful therapy with low patient compliance. These drawbacks can be overcome by producing a smart capsule with a blood glucose concentration-sensitive envelope that can transition from off to on level for insulin release governed by glucose concentrations [127].

A classic method to produce smart capsules is to use stimuli-responsive polymeric materials embedded on the pores or surfaces of porous membranes as smart on/off switches [131]. Polymers could be introduced onto porous membranes using “grafting to” and “grafting from” methods.

For the “grafting from” method, there is pre-activation of polymerization sites on the membranes through chemical agents, UV light, plasma or heat; on these activated sites the functional monomers “grow” and form smart gates. For the grafting-to method, polymerization of functional monomers occurs before grafting onto the porous membrane.

The drug release scheme predicts that in the absence of the predetermined specific stimulus, the gate remains closed and thus there is no release. Under the action of the stimulus, the gate opens, and drug release occurs [125].

Smart capsules can be developed using hydrogel as the membrane of the entire capsule. Among the membrane modeling methods, the microfluidic technique comes up with special features of precision in manipulating the shape, structure and composition of monodisperse emulsion droplets [132].

Zhang et al. [133] obtained, through a plate-emulsion microfluidic methodology, a system encapsulated in a glucose-reactive hydrogel. 3-Acrylamidophenylboronic acid (AAPBA) was used as the glucose sensor, and thermoresponsive poly(N-isopropylacrylamide) (PNIPAM) was used as the activator. In the range of physiological blood glucose concentration at 37 °C, the capsule showed a reversible and repeated swelling/shrinking response. The system developed by Zhang and coworkers thus provides a promising model to produce smart drug delivery systems.

Implantable systems that can release drugs in a programmable mode according to therapeutic needs also have a viable use in cancer treatment. Wang et al. [134] fabricated a composite membrane by incorporating both pH and temperature-sensitive hydrogel microparticles with magnetic silk fibroin nanoparticles inside. The application of an alternating magnetic field with the subsequent generation of heat by the magnetic nanoparticles led to the contraction and swelling of the microgels. This induced a reversible change in membrane permeability that allowed immediate release of the model drug Rhodamine B (Rh.B). By adjusting the thickness of the membrane and the ratio of the amount of microgel to the number of magnetic nanoparticles, control of the release rate could be achieved.

The release rate of Rh.B is increased under acidic conditions compared with its value at physiological pH. It is well known that oncological diseases lead to a lowering of pH, and thus this experimental observation has prospected a relevant potential for the use of the membrane in selective cancer therapy.

Still for the development of an oncology drug delivery system, more particularly for breast and liver cancer, a membrane of polyvinyl/cellulose nanocrystals (PVA/CNCs) loaded with curcumin [135] has been made. The strategy to maximize the encapsulation capacity of the hydrogel was directed toward finding an optimal preparation method. This was found in the solution fusion method using citric acid as a crosslinker. FT-IR spectroscopy revealed that curcumin and membrane components are bound through an intermolecular hydrogen bond in the amorphous phase of the PVA/CNC system. Curcumin was released in bursts (41%) during the first hour, after which a sustained release of 70% and 94% was shown in 24 h and 48 h, respectively.

Kamoun et al. [136] developed hydrogel membranes based on hyaluronic acid (HA) and poly(N-isopropylacrylamide) sensitive to pH and temperature. Such membranes were produced by redox polymerization, using N,N-methylenbisacrylamide (BIS) and epichlorohydrin (EPI) as crosslinkers. The membranes were loaded with ampicillin antimicrobial drug, and it was observed that, as the ratios of HA varied, the swelling capacity and release rate varied too. In addition, the reactivity to heat and pH allowed a rapid release. Thus, this intelligent system could be used for rapid drug release to different districts.

In 2016, Nagarjuna et al. [137] developed a hydrogel membrane consisting of a mixture of sodium alginate (SA) and Karaya rubber (KG). It was used for testing the sustained release, in physiological conditions obtained by phosphate buffer (pH 7.4 T 37 °C), of flutamide (FLT), a potent nonsteroidal antiandrogenic prostate anticancer drug, which was membrane-loaded. The results showed a decreased swelling with increasing KG amount. FT-IR spectroscopical analysis confirmed the absence of chemical interactions between drug and polymer. Results of controlled release tests showed that the amount of the released flutamide increased with the amount of SA in the membrane.

## 9. Conclusions and Perspectives

Relevant aspects concerning the use of hydrogel membranes in the field of biomedical applications have been considered in this review. Some lines of research are an interesting challenge especially for future development. It is these, that our attention has been focused on and it is precisely the most innovative studies that we have decided to report.

Regarding the restoration of liver function, one of the most interesting research directions concerns decellularized ECM-based hydrogel materials [41]. It has been seen how decellularized liver matrix coating on 3D cryogels can promote hepatocyte growth and function in vitro. This is really a very important aspect that for the future can be proposed as a viable alternative to the surgical transplantation approach. Another interesting perspective is that offered by miniaturized bioreactors, in which cells reach a 3D arrangement and are cultured under dynamic conditions. They have been proposed as alternative cell culture models and have become popular as “organs-on-a-chip” [42,46,47,48]. The encapsulation of cells within a three-dimensional (3D) membrane of hydrogels that mimics the supporting functions of the extracellular matrix (ECM), prolongs hepatic cell functions.

As far as pancreatic function is concerned, the most promising aspects of research involve biomimetic microencapsulation of beta islets. The best results have been obtained from natural hydrogels, such as those based on alginate and ECM, cross-linked through ionic gelation, but other systems may be explored in the future.

Numerous studies have shown that zwitterionic polymers are ideal candidates for preventing thrombosis and infection thanks to their super hydrophilic properties. These characteristics, however, weaken their mechanical properties, which can be restored by introducing reinforcing materials [73,75], such as poly (carboxy betaine) (pCBM) microgels. In the future, inspired by this type of integration, hydrogel membranes may be supplemented with stabilizing molecules, having also antibacterial and antithrombotic functions.

The introduction of metal nanoparticles within hydrogel membranes and, more particularly, of silver metal nanoparticles, has shown great efficacy, and thus the great possibility for future development, in promoting wound healing, acting for a sufficiently long time, and preventing degradation of bioactive polymers.

In recent years. the use of porous and/or biodegradable microspheres together with the use of 3D printing has been presented as a great resource, for example, to make great strides in bone and cartilage tissue engineering. The resulting porous hydrogel can be used in two ways: (a) as carrier of cells that go on to promote tissue regeneration; (b) as a constituent of permanent implants to replace damaged cartilage tissue [109].

Regarding the development of hydrogel membranes for drug delivery, recently, the prospect of producing implantable systems that can release drugs in a programmable mode, according to therapeutic needs and minimize the risk of side effects, has opened new horizons to research [129,130]. This controlled release can be achieved by producing smart composite membranes that incorporate hydrogel microparticles that are sensitive to pH and/or temperature and/or magnetic fields. For example, the application of an alternating magnetic field to a hydrogel membrane containing magnetic silk fibroin nanoparticles [134] can generate heat from the magnetic nanoparticles, and thus a reversible change in membrane permeability that inducing the release.

## Data Availability

Not applicable.
